# Practical Notes and Formulæ

**Published:** 1879-01-20

**Authors:** 


### PRACTICAL NOTES AND FORMULÆ.

**Salicylic Acid in Scarlet Fever and Diphtheria.**—Dr. Pownall, in *British Medical Journal*, says : For the last three years I have used, with unvarying success, the salicylic acid suspended in mucilage in both mild and severe forms of scarlet fever, and have seen the throat-symptoms and fever rapidly abate and the patients make rapid recoveries. On being called to a case, I have given doses varying from five to ten grains every two hours until the throat-symptoms and fever abated, and find that little patients for whom we can do so little, when obliged to use the mop or brush to the throat, experience no inconvenience in taking this medicine, which, being simply in a state of suspension, has a chance of (at least some portion of it) remaining on the throat, and so acting as a topical remedy, whilst the remainder acts as an invaluable antipyretic. The success in cases of scarlet fever has led me to try the same remedy for diphtheria ; and I am happy to say that in the most virulent cases of diphtheria, I have seen the pellicle broken up and the diphtheritic patch removed in a most marvelous manner. Indeed, since the use of salicylic acid in diphtheria, I have not seen one fatal case, although several were of a very dangerous type. It is but fair to say that, in diphtheria, my mode of action is giving the salicylic every four hours, and tinctura ferri perchloridi (*P. B.*) alternately with it.

I append the form I use:

℞ Acidi salicylici.................. ʒ i. vel. ʒ ij.

Syrupi simplicis.......................... ʒ iv.

Mucilaginis tragacanthi................ ℥ i.

Tincturæ aurantii......................... ʒ iv.

Aquæ q. s. ad.............................. ℥ vi.

Fiat mistura. Capiat ʒ iv 2dis horris.

**Treatment of Deep Sinuses by Villate's Mixture.**—Several deep sinuses have recently been under treatment in the surgical service in which no necrosed bone could be found, but which proved intractable to heal. Villate's mixture was tried first of half strength, then of full strength. In some of the cases it proved of value, in others it failed partially or completely. The case in which it proved of most service was one of deep sinuses in the neighborhood of the hip-joint. The original composition of the mixture was :

℞ Liq. plumbi subacet.,................ ℥ j.

Zinci sulph. cryst.,.................

Cupri sulph. cryst.,.... ........aa ʒ ss.

Aceti vini albi,................. fl. ℥ vjss.

This mixiure was injected once a day, and proved a more satisfactory application than any other. Some patients complained of severe pain, others felt but slight inconvenience from it.—*New York Medical Journal.*

**Diabetes.**—Ammonium phosphate has heen found useful in diabetes. It has been used in 18 gr. doses three times per day. The sugar disappears from the urine, and the strength of the patient rapidly returns.

**Toothache Drops.**—Mr. J. Merson, L. D. S., states, in the *British Journal of Dental Science*, that acute pain can be often suppressed by pungent aromatics, just as we know essential oils are popular remedies for tooth-ache, as are creasote, peppers, spirits, etc. But better still, he tells us that combined with chloroform and aconite, they will prevent the pain of tooth extraction. Hundreds of patients told him they did not feel the pain. Here is his formula for a local anæsthetic to supercede chloroform, ether, etc.

| | | |
|---|---|---|
| ℞ Chloroform pur | ʒ iij. | |
| Tr. aconiti | ʒ iij. | |
| Tr. capsici | ʒ j. | |
| Tr. pyrethri | ʒ ss. | |
| Ol. caryoph | ʒ ss. | |
| Gum camph | ʒ ss. | M. |

The tooth and surrounding gums are to be previously dried, and four or five drops of this applied with cotton wool. Then without delay use the forceps, but the instrument *must be warmed.* This is most important. We have felt the pang of the cold steel, and, whether the anæsthetic or not be used, agree as to the propriety of using warm instruments. For toothache, a pellet of cotton wool soaked in the above may be introduced into the cavity, and is said often to give speedy relief.—*Boston Journal of Chemistry.*

**Anti-emetics.**—

℞ Subnitrate of bismuth.... gr v

Give every half hour. Particularly suited to nausea attending the diarrhœas of children.

**Carbolic Acid as an Anti-emetic.**—The formula of Prof. Davis, of Chicago, for using carbolic acid as an anti-emetic is the following:

| | | |
|---|---|---|
| ℞ Crystal. carbolic acid | grs iij | |
| Glycerine | ℥ ss | |
| Tinc. opii | " j | |
| Water | " ss | M |

Dose, twenty drops every half hour until vomiting ceases, then every two hours.

**Ipecac as an Anti-emetic.**—

| | | |
|---|---|---|
| ℞ Fluid ext. ipecac | gtt x | |
| Water | ℥ ij | M |

Dose, one teaspoonful every half hour.

**Calomel as an Anti-emetic.**—

℞ Calomel.................. gr ij

Divide into eight powders, sprinkle one on the tongue every half hour for a few doses.

**Podophylin.**—Dr. Loyd, at Pharmaceutical Association, said: That as a rule the whiter the color of podophylin the better. All shades can be made and yet be pure; the first percolate produces a darker podophylin than a later one.

THE following was handed us by a friend who has long used it successfully in his family:

**Relief for Dyspepsia.**—

| | | |
|---|---|---|
| ℞ | Oil peppermint.................... | 10 drops |
| | White sugar...................... | 1 drachm |
| | Pulverized rhei .................. | 2 " |
| | Soda (pure)....................... | 2 " |
| | Water............................ | 6 ounces |
| | Brandy .......................... | 2 " |

Rub oil peppermint and sugar first together, then add other ingredients.

Dose : Adults, one tablespoonful after meals ; children, half to one teaspoonful.

Should the prescribed dose disturb the bowels too much, lessen it; only take enough to correct the stomach, which it will do almost instantly.

**The Use of Ipecac in Labor.**—Dr. J. H. Carriger, of Knoxville, Tenn., (*New York Medical Journal*), says: I believe it will at last be conceded that we have in ipecacuanha an oxytocic, potent, and safer than ergot for both mother and child, because it stimulates the uterus into a more nearly normal action, and at the same time facilitates dilation of the rigid os, a circumstance making it useful in a large number of cases which would otherwise be slow and tedious, and in which the endurance of the patient is liable to become exhausted by prolonged and efficient pains. In cases of this kind I have found it prompt in promoting dilation, prompt in changing the character of the pains, when they were of the cutting, sawing, and inefficient kind, rendering them forcible, expulsive, and at the same time much more bearable than they had previously been, and thus hastening the period of convalescence and of safety.

[He gives two grains at a dose, and repeats if necessary.—EDS.]

**Perchloride of Iron as a Topical Application for Chancre.**—In an article on iron, in the *Dictionnaire Encyclopedique des Sciences Médicales* M. Rollet gives the following formulæ for topical use in cases of chancre :

| | | | | |
|---|---|---|---|---|
| ℞ | Aquæ............ | ʒ vi. | (24 grammes). | |
| | Ferri perchloridi.. | ʒ iij. | (12 " ). | |
| | Acidi citrici...... | ʒ i. | ( 4 " ). | M |

And :

| | | | | |
|---|---|---|---|---|
| ℞ | Acidi hydrochlor. } | | | |
| | Acidi citrici........ } aa | ʒ i. | ( 4 grammes). | |
| | Ferri perchloridi. } | | | |
| | Aquæ distillatæ...... | ℥ i. | (32 " ). | M |

**Dysmenorrhœa**—The following formula has been recommended as peculiarly adapted to cases of dysmenorrhœa dependent upon nervous hyperesthesia with a contraction of muscular fiber:

| | | | |
|---|---|---|---|
| ℞ | Tinc. macrotys. | | |
| | Tinc. pulsatilla.......................aa | ʒ ss | |
| | Water.................................. | ℥ iv | M |

A teaspoonful three or four times a day, commencing a week before the expected period, and continuing until it is fully established.

**Suggestons for Treating Swollen Fingers.**—A writer to the *Medical Times*, London, says: For two or three years past I have used a piece of India-rubber finger-stall in fissures and slight cuts of the fingers; and for twelve months or more I have used it in cases of thickening or deposit around the joints of the fingers after injury, with great relief to the patient. It has seemed to me that the brown finger-stalls of pure rubber are better than the black or vulcanized.

A piece of tubing may be cut into lengths of about an inch or an inch and a half. One of these can be slipped over the joint by the patient himself, after he has been taught how to do it. It should be worn constantly, day and night. The patient will soon learn how to roll it off, and reapply it after washing his hands. When it has become too loose to give the necessary support, another length can be taken.

**Neuralgia.**—In a case of intense neuralgia in a delicate female, in the eye and temporal region, in which the patient had an idiosyncrasy against opiates, the following prescription gave relief in two hours. Apply chloroform locally as a revulsive, and:

| | | | |
|---|---|---|---|
| ℞ | Tinc. aconit............................ | gtts xij | |
| | Bromide of ammonium............... | ʒj | |
| | Camphor water......................... | ℥j | M |

Give one third, or about a tablespoonful every hour.

**Carya Oliviformis** (*Pecan nut*).—Dr. W. D. Hunt, of Kentucky, writes:

"Having cases demanding astringents in their treatment, and none at hand, I was reminded of the peculiar astringent property of the intra lining of the fruit of the carya oliviformis (pecan nut), from which I prepared a tincture. Having tried it, I found it to be almost a *sine qua non* in the treatment of any inflammatory condition of the fauces; also in diarrhœa, especially that of infants, as an adjuvant to the chalk mixture."

**Rheumatism in Scrofulous Subjects.**—

| | | | |
|---|---|---|---|
| ℞ | Com. tinc. cinchona....................... | ℥vj | |
| | Tinc. phitolacca............................ | ℥iij | |
| | Iodide potassium........................... | ʒss | M |

Dose a teaspoonful three or four times per day. Useful also in nocturnal syphilitic pains, and in the eruptive affections of both secondary and tertiary syphilis.

**Cascara Sagrado, etc.**—It is affirmed that *Cascara Sagrado* and the plant known as *Rhamus Purskiana* are one and the same thing; that *yerba reuma* and *frankenia grandifolia* are the same; and that the *Mountain* or *Oregon Grape*, of California, and the *Berberis aquifolium* are identical.

**Hot Water and Creosote as an Anti-emetic.**—In some cases of bilious vomiting wherein the stomach has been thoroughly emptied, we have stopped the most obstinate vomiting by the application of mustard to the epigastrium, and administering a glass of water as hot as it could be borne containing one drop of creosote.

